# Device and surgical procedure-related infections in Canadian acute care hospitals, 2020–2024

**DOI:** 10.14745/ccdr.v52i05a04

**Published:** 2026-05-31

**Authors:** Canadian Nosocomial Infection Surveillance Program

**Affiliations:** 1 Centre for Communicable Diseases and Infection Control, Public Health Agency of Canada, Ottawa, ON

**Keywords:** hospital-associated infection, acute care, surveillance, antimicrobial resistance, device-associated infection, surgical procedure-related infection, surgical site infection, ICU-CLABSI, central line-associated bloodstream infection, hip and knee arthroplasty surgical site infection, cerebrospinal fluid shunt surgical site infection, paediatric cardiac surgical site infection, Canada

## Abstract

**Background:**

Healthcare-associated infections (HAIs) are a significant healthcare burden in Canada. National surveillance of HAIs at sentinel acute care hospitals is conducted by the Canadian Nosocomial Infection Surveillance Program.

**Objective:**

To describe device and surgical procedure-related HAI epidemiology in Canada from 2020 to 2024.

**Methods:**

Data were collected from up to 67 Canadian sentinel acute care hospitals between January 1, 2020 and December 31, 2024 for intensive care unit central line-associated bloodstream infections (ICU-CLABSIs), hip and knee surgical site infections (SSIs), cerebrospinal fluid (CSF) shunt SSIs and paediatric cardiac SSIs. Case counts, rates, patient and hospital characteristics, pathogen distributions and antimicrobial resistance data are presented.

**Results:**

Between 2020 and 2024, 1,846 device-related infections and 1,014 surgical procedure-related infections were reported. Rates of ICU-CLABSIs, hip and knee SSIs, CSF shunt SSIs and paediatric cardiac SSIs fluctuated throughout the study period, with no significant trends observed. The most commonly identified pathogens were coagulase-negative staphylococci (37%) in ICU-CLABSIs and *Staphylococcus aureus* (41%) in SSIs.

**Conclusion:**

Epidemiological and microbiological trends among selected device and surgical procedure-related HAIs are essential for benchmarking infection rates nationally and internationally, identifying any changes in infection rates or antimicrobial resistance patterns and helping inform hospital infection prevention and control and antimicrobial stewardship policies and programs.

## Introduction

Healthcare-associated infections (HAIs) are a common outcome of healthcare delivery and among hospitalized patients prolong hospital stays and require additional treatment (1,2). Healthcare-associated infections can result from the use of invasive medical devices and surgical procedures (3) and are commonly reported in Canadian hospitals, where they are significantly associated with hospital readmissions and all-cause mortality, compared with surgical patients without an associated infection (4).

A point prevalence study conducted in 2024 in Canadian sentinel acute care hospitals revealed that one third (33%) of all reported HAIs were device associated (5). Among all adult inpatients, the prevalence of surgical site infections (SSIs) in this study was 1.6% and the prevalence of central line-associated bloodstream infections (CLABSIs) was 0.7% (5). The risk of device and surgical procedure-related infections is associated with factors including patient demographics, prior surgeries and the duration of hospital stay, in addition to the type of hospital in which the patient received care (6–8).

Understanding the epidemiology of HAIs related to medical devices and surgical procedures is crucial for establishing benchmark rates over time. These benchmarks support the development of effective antimicrobial stewardship programs and guide infection prevention and control strategies. Collecting and analyzing antimicrobial susceptibility data are crucial for guiding appropriate antimicrobial use and combating antimicrobial resistance (9). This report presents an epidemiological summary of specific device and surgical procedure-related HAIs reported between 2020 and 2024 across 67 hospitals participating in the Canadian Nosocomial Infection Surveillance Program (CNISP).

## Methods

### Design

Since its establishment in 1994, CNISP has conducted national HAI surveillance at sentinel acute care hospitals across Canada, in collaboration with the Public Health Agency of Canada and the Association of Medical Microbiology and Infectious Disease Canada. Data are presented for device-related infections including intensive care unit (ICU)-CLABSIs, and surgical procedure-related HAIs including hip and knee arthroplasty SSIs, cerebrospinal fluid (CSF) shunt SSIs, and paediatric cardiac SSIs.

### Case definitions

Device and surgical procedure-related HAIs were defined according to standardized protocols and case definitions (see **Appendix**). Complex infections, defined as deep incisional and organ/space, were included in hip and knee SSI surveillance. Central line-associated bloodstream infections identified in ICU settings were included in CLABSI surveillance. The adult mixed patient ICU, adult cardiovascular surgery intensive care unit (CVICU), paediatric intensive care unit (PICU) and neonatal intensive care unit (NICU) were included as eligible ICU settings. Adult mixed ICUs included any adult ICU with a mix of patient types as part of the ICU patient mix (i.e., medical/surgical, surgical/trauma, burn/trauma, medical/neurosurgical).

### Data source

Epidemiological data for device and surgical procedure-related infections identified between January 1, 2020 and December 31, 2024 (using surgery date for SSIs and date of positive blood culture for CLABSIs) were submitted by participating hospitals using standardized data collection forms. Hospital participation varied by surveillance project and year. Data submission and case identification were supported by training sessions and periodic evaluations of data quality.

### Statistical analysis

To calculate hip and knee SSI, CSF shunt SSI and paediatric cardiac SSI rates, the number of cases were divided by the number of surgical procedures performed (multiplied by 100). To calculate ICU-CLABSI rates, the number of cases was divided by line day denominators (multiplied by 1,000). Neonatal ICU-CLABSI rates were also calculated per 1,000 line days by birth weight category (750 g or less, 751 g–1,000 g, 1,001 g–1,500 g, 1,501 g–2,500 g and more than 2,500 g). To calculate ICU-specific catheter utilization, the total number of ICU patient central line days was divided by the total number of ICU patient days. To calculate proportions of pathogens, the number of pathogens were divided by the total number of identified pathogens. Denominators may vary, as missing and incomplete data were excluded from analyses. Median and interquartile ranges (IQRs) were calculated for continuous variables. Trends over time were tested using the Mann-Kendall test. The chi-square test was used to compare two categorical variables. Significance testing was two-tailed, and differences were considered significant at a *p*-value of ≤0.05. Analyses were conducted using R version 4.4.3.

## Results

Between 2020 and 2024, up to 67 unique hospitals submitted device and surgical procedure-related infection data to CNISP. In the most recent year of surveillance data available, 2024, 67 hospitals submitted these data (**Table 1**), with the majority of participating hospitals located in the Western (British Columbia, Alberta, Manitoba and Saskatchewan; n=29, 43.3%) and Central (Ontario and Québec; n=28, 41.8%) regions. Additionally, the majority of hospitals served an adult-only (n=26, 38.8%) or mixed adult-paediatric (n=22, 32.8%) population and were medium-sized (201–499 beds; n=29, 43.3%) (Table 1). Overall, 1,846 ICU-CLABSIs and 1,014 surgical procedure-related infections were reported (**Table 2**) between 2020 and 2024. Among all SSIs reported, hip and knee infections represented 70.2% (n=712) of these types of infections (Table 2).

**Table 1:** Characteristics of acute care hospitals participating in device and surgical procedure-related healthcare-associated infection surveillance, 2024

Characteristic of hospitals	CLABSI-adult mixed ICU	CLABSI-adult CVICU	CLABSI-PICU	CLABSI-NICU	CSF shunt SSI	Paediatric cardiac SSI	Hip and knee SSI	Total unique hospitals
Total number of participating hospitals	40	9	12	20	11	6	30	67
**Region^a^**								
Western	17	4	5	8	4	1	16	29
Central	19	4	6	9	5	4	7	28
Eastern	4	1	1	3	2	1	7	10
Northern	N/A	N/A	N/A	N/A	N/A	N/A	N/A	N/A
**Hospital type**								
Adult^b^	22	4	N/A	N/A	2	N/A	12	26
Adult-NICU	6	2	N/A	3	N/A	N/A	2	6
Mixed^c^	12	3	1	5	2	N/A	16	22
Paediatric^d^	N/A	N/A	8	8	7	4	N/A	99
Paediatric-OB	N/A	N/A	3	4	N/A	2	N/A	4
**Hospital size**								
Small (1–200 beds)	4	1	7	9	5	4	6	19
Medium (201–499 beds)	20	3	4	7	3	2	14	29
Large (500 and more beds)	16	5	1	4	3	N/A	10	19

Abbreviations: CLABSI, central line-associated bloodstream infection; CSF, cerebrospinal fluid; CVICU, cardiovascular surgery intensive care unit; ICU, intensive care unit; N/A, not applicable; NICU, neonatal intensive care unit; PICU, paediatric intensive care unit; SSI, surgical site infection

^a^ Western region includes British Columbia, Alberta, Manitoba and Saskatchewan; Central region includes Ontario and Québec; Eastern region includes New Brunswick, Nova Scotia, Prince Edward Island and Newfoundland and Labrador; Northern region includes Yukon, Northwest Territories and Nunavut

^b^ Adult standalone hospitals excluding adult facilities with a neonatal intensive care unit

^c^ Mixed hospitals provide both adult and paediatric care

^d^ Paediatric standalone hospitals excluding mixed facilities with women’s and obstetric wards

**Table 2:** Number of device and surgical procedure-related healthcare-associated infection, by type and year, 2020–2024

Infection type	2020	2021	2022	2023	2024	Total 2020–2024
ICU-CLABSI	273	416	356	435	366	1,846
CSF shunt SSI	22	20	28	14	19	103
Paediatric cardiac SSI	37	35	25	52	50	199
Hip and knee SSI	85	126	166	165	170	712
Total infections	417	597	575	666	605	2,860

Abbreviations: CLABSI, central line-associated bloodstream infections; CSF, cerebrospinal fluid; ICU, intensive care unit; SSI, surgical site infection

A total of 3,111 pathogens were identified from device-related infections and 1,072 pathogens from surgical procedure-related cases between 2020 and 2024 (**Table 3**). Of the identified pathogens for ICU-CLABSIs, 59.6% were gram-positive, 24.7% were gram-negative and 15.7% were fungal. Coagulase-negative staphylococci (CoNS) and *Enterococcus* spp. were most frequently identified in cases of ICU-CLABSIs. Of the identified pathogens for SSIs, 79.6% were gram-positive, 19.1% were gram-negative and 1.3% were fungal. Coagulase-negative staphylococci and *Staphylococcus aureus* were the most common pathogens associated with SSIs (Table 3). From 2020 to 2024, the proportion of *S. aureus* that was methicillin-resistant (MRSA) was 16.7% for ICU-CLABSIs and 9.9% for SSIs (data available on request).

**Table 3:** Distribution and rank of the most frequently reported gram-negative, gram-positive and fungal pathogens, 2020–2024^a^

Pathogen category	Rank	Pathogen	ICU-CLABSI		Hip and knee		CSF shunt		Paediatric cardiac	
			N=3,111		N=805		N=111		N=156	
			n	%	n	%	n	%	N	%
Gram-positive	1	Coagulase-negative staphylococci^b^	678	21.8	139	17.3	33	29.7	22	14.1
	2	*Enterococcus* spp.	666	21.4	50	6.2	2	1.8	1	0.6
	3	*Staphylococcus aureus* ^c^	305	9.8	315	39.1	32	28.8	92	59.0
	4	*Streptococcus* spp.	65	2.1	70	8.7	4	3.6	11	7.1
	Other gram-positive^d^		139	4.5	59	7.3	15	13.5	8	5.1
	Total gram-positive		1,853	59.6	633	78.6	86	77.5	134	85.9
Gram-negative	1	*Klebsiella* spp.	173	5.6	14	1.7	8	7.2	4	2.6
	2	*Escherichia coli*	132	4.2	25	3.1	3	2.7	0	0.0
	3	*Enterobacter* spp.	129	4.1	34	4.2	4	3.6	6	3.9
	4	*Pseudomonas* spp.	95	3.1	28	3.5	3	2.7	0	0.0
	5	*Serratia* spp.	58	1.9	14	1.7	2	1.8	2	1.3
	Other gram-negative^e^		182	5.9	52	6.5	3	2.7	3	1.9
	Total gram-negative		769	24.7	167	20.7	23	20.7	15	9.6
Fungi	1	*Candida albicans*	259	8.3	4	0.5	0	0.0	0	0.0
	2	Other *Candida* spp.^f^	215	6.9	1	0.1	2	0.9	7	4.5
	Other fungi^g^		15	0.5	0	0.0	0	0.0	0	0.0
	Total fungal		489	15.7	5	0.6	2	1.8	7	0.0
Total			3,111	100	805	100	111	100	156	100

Abbreviations: CLABSI, central line-associated bloodstream infections; CSF, cerebrospinal fluid; ICU, intensive care unit

^a^ The number of pathogens may not equal the number of infections as each case of device-related and surgical site infection may include multiple pathogens

^b^ Coagulase-negative staphylococci included *S. lugdunensis*, *S. haemolyticus*, *S. epidermidis*, *S. capitis*, *S. hominis* and *S. warneri*

^c^ *Staphylococcus aureus* includes methicillin-resistant *S. aureus*, methicillin-susceptible *S. aureus* and unspecified *S. aureus*

^d^ Other gram-positive pathogens included anaerobic gram-positive cocci, *Finegoldia magna*, *Clostridioides* spp., *Lactobacillus* spp. and others

^e^ Other gram-negative pathogens included *Stenotrophomonas* spp., *Morganella morganii*, *Proteus mirabilis*, *Pantoea* spp., *Prevotella* spp., *Bacteroides fragilis* and others

^f^ Other *Candida* spp. included *C. dubliniensis*, *C. glabrata*, *C. krusei*, *C. lusitaniae*, *C. parapsilosis* and *C. tropicalis*

^g^ Other fungi included *Aspergillus* spp., *Trichophyton tonsurans* and unspecified fungi

### Intensive care unit central line-associated bloodstream infections

**Infection characteristics:** Between 2020 and 2024, a total of 2,801 CLABSIs were reported. Most infections occurred in adult mixed ICUs (65.9%, n=1,846) and NICUs (17.3%, n=484), reflecting higher site participation in CLABSI surveillance in these ICU settings. Patient demographics and outcomes for ICU-related CLABSIs are summarized in **Table 4**. Among patients with CLABSIs in adult ICUs, the median age was older in the adult CVICU compared to adult mixed ICUs (*p*<0.001). Across all ICU settings, the majority of those with CLABSIs were male, ranging from 57.4% in the PICU to 68.2% in the adult CVICU. The median time from ICU admission to infection was longest in the PICU (28 days, IQR: 12−66 days) while shorter time periods were reported in all other ICU settings, ranging from 10−14 days (*p*<0.001).

**Table 4:** Patient characteristics and outcomes of intensive care unit central line-associated bloodstream infections, 2020–2024

Characteristic	Adult mixed ICU (N=1,846)	Adult CVICU (N=182)	PICU (N=289)	NICU (N=484)
Age, median (IQR)	59 years (46 years–68 years)	65 years (52 years–72 years)	6 months (3 months–24 months)	21 days (9 days–52 days)
Sex, female/total (%)	582 (31.5%)	55 (30.2%)	123 (42.6%)	184 (38.0%)
Birthweight (g), median (IQR)	N/A	N/A	N/A	947 (IQR: 669–2,130)
Gestational age (weeks), median (IQR)	N/A	N/A	N/A	27.0 (IQR: 24.1–34.0)
Days from ICU admission to infection, median (IQR)	11 (IQR: 6–21)	10 (IQR: 6–18)	28 (IQR: 12–66)	15 (IQR: 8–38)
Deaths, thirty-day all cause (%)	606 (32.9%)	58 (31.9%)	28 (9.7%)	51 (10.6%)

Abbreviations: CVICU, cardiovascular surgery intensive care unit; ICU, intensive care unit; IQR, interquartile ranges; NICU, neonatal intensive care unit; N/A, not applicable; PICU, paediatric intensive care unit

**Figure 1:**
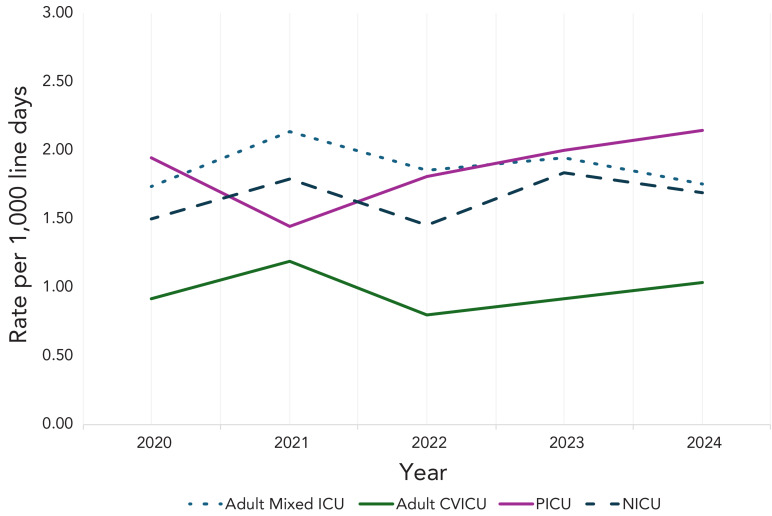
Rate of central line-associated bloodstream infection per 1,000 line days by intensive care unit type, 2020–2024 Abbreviations: CVICU, cardiovascular intensive care unit; ICU, intensive care unit; NICU, neonatal intensive care unit; PICU, paediatric intensive care unit

**Trends over time:** From 2020 to 2024, adult mixed ICUs had the highest overall CLABSI rates (1.89 infections per 1,000 line days), followed by PICUs (1.88 infections per 1,000 line days), NICUs (1.66 infections per 1,000 line days) and adult CVICUs (0.97 infections per 1,000 line days) (Appendix, **Table A1**). From 2020 to 2024 in adult ICU settings, CLABSIs rates fluctuated for adult mixed ICUs (1.74–1.82 infections per 1,000 line days, *p*=0.45) and adult CVICUs (0.80–1.19 infections per 1,000 line days, *p*=0.57) (**Figure 1**). Both adult mixed ICU CLABSI and adult CVICU rates peaked in 2021 with a rate of 2.14 and 1.27 infections per 1,000 line days, respectively. Catheter utilization from 2020 to 2024 ranged from 70.1%–74.2% in adult mixed ICUs and 66.4%–87.0% in adult CVICUs (data available on request).

**Figure 2:**
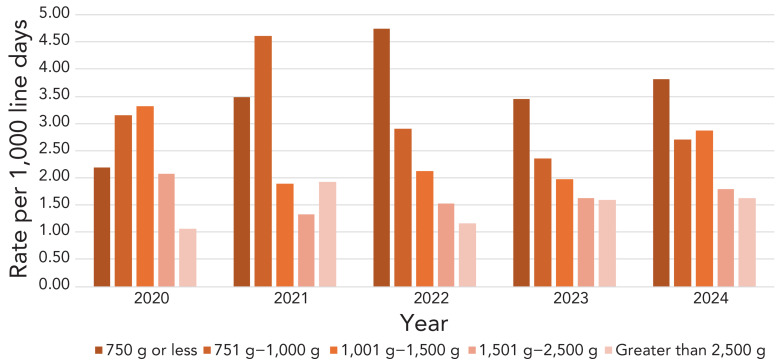
Rate of neonatal intensive care unit central line-associated bloodstream infections per 1,000 line days by birthweight, 2020–2024

In paediatric ICUs, NICU and PICU CLABSIs fluctuated from 2020 to 2024, with NICU CLABSI rates ranging between 1.46 to 1.84 infections per 1,000 line days (Figure 1). In addition, PICU CLABSIs were lowest in 2021 (1.45 infections per 1,000 lines days), followed by increases in each year from 2022 to 2024 (Figure 1). Of the 65.7% (n=318/484) of NICU CLABSI cases from participating sites with birthweight-specific data, rates of CLABSIs in the NICU per 1,000 line days were highest among the infants with lower birthweight (1,000 g or less) from 2020 to 2024, peaking in 2022 for 750 g or less (4.75 infections per 1,000 lines days), with rates generally decreasing as birthweight increased (**Figure 2**). Catheter utilization in PICUs ranged from 58.9%–66.6% from 2020 to 2024 while NICU had the lowest catheter utilization overall during the same time period, ranging from 28.1%–29.5% (data available on request).

All-cause mortality at thirty days was highest in the adult mixed ICU and adult CVICU at 32.8% and 31.9%, respectively, while thirty-day all-cause mortality ranged from 9.7%–10.6% in paediatric and neonatal ICU settings. The most commonly identified pathogens among ICU-CLABSIs overall were CoNS (21.8%) and *Enterococcus* spp. (21.4%), which aligned with the most commonly identified pathogens among adult mixed ICUs and adult CVICUs. Among PICU and NICU CLABSIs, the most commonly identified pathogens were CoNS and *S. aureus* (data available on request).

### Hip and knee surgical site infections

**Infection characteristics:** Between 2020 and 2024, a total of 712 complex hip and knee SSIs were reported, with hip arthroplasties accounting for most of the cases (n=440, 61.8%). Among these SSIs, 51.8% (n=369) were organ/space infections, while 48.2% (n=343) were deep incisional infections (**Table 5**). The median patient age was 69 years (IQR: 60–77 years) for hip SSIs and 67 years (IQR: 61–7 years) for knee SSIs. The median time from procedure to infection onset was 23 days (IQR: 16–36 days) for hip SSIs and 24 days (IQR: 18–37 days) for knee SSIs. The median length of stay was two days for hip (IQR: 1–7.5 days) and one day for knee (IQR: 1–3 days) SSIs.

**Table 5:** Frequency of hip and knee surgical site infections by year and infection type, 2020–2024

Year	Deep incisional SSI			Organ/space SSI		All cases
	n	%		n	%	n
**Hip arthroplasty**						
2020	22		45.8	26	54.2	48
2021	44		49.4	45	50.6	89
2022	48		46.2	56	53.9	104
2023	47		52.2	43	47.8	90
2024	54		49.5	55	50.5	109
Overall	215		48.9	225	51.1	440
**Knee arthroplasty**						
2020	14		37.8	23	62.2	37
2021	23		62.2	14	37.8	37
2022	34		54.8	28	45.2	62
2023	34		45.3	41	54.7	75
2024	23		37.7	38	62.3	61
Overall	128		47.1	144	52.9	272

Abbreviation: SSI, surgical site infection

**Figure 3:**
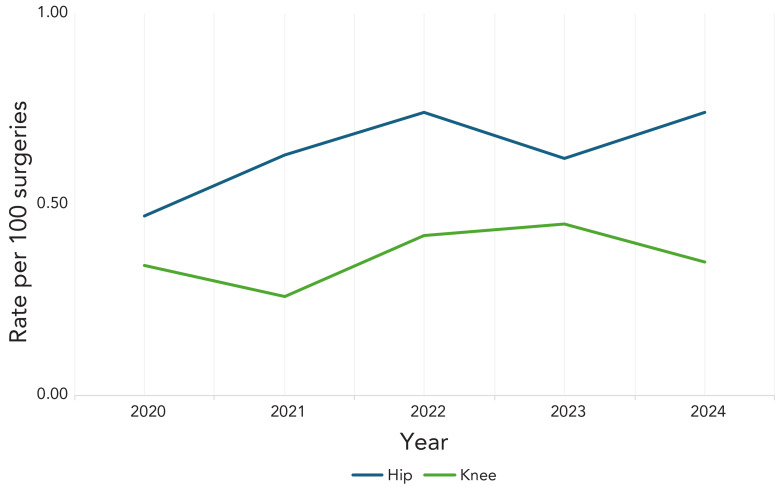
Rate of hip and knee surgical site infections per 100 surgeries, 2020–2024

**Trends over time:** Between 2020 and 2023, knee SSI rates increased non-significantly by 32.4% (0.34–0.45 infections per 100 surgeries, *p*=0.31), before decreasing to a rate of 0.35 infections per 100 surgeries in 2024. Hip SSI rates fluctuated between 0.47 and 0.74 infections per 100 surgeries (*p*=0.31) (**Figure 3**; Appendix, **Table A2**). Most patients (74.0%, n=527/712) with a hip or knee SSI were readmitted and 65.3% (n=465/712) required revision surgery. Within 30 days after the first positive culture, 15 all-cause deaths (3.5%, n=15/440) were reported among patients with a complex SSI following a hip arthroplasty, while no deaths were reported among knee arthroplasty SSI patients. The most common pathogens identified among hip and knee SSIs were *S. aureus* (39.1%) and CoNS (17.3%) (Table 3), with no significant differences by infection type.

### Cerebrospinal fluid shunt surgical site infections

**Infection characteristics:** Between 2020 and 2024, a total of 103 CSF shunt SSIs were reported. The median patient age was 49 years (IQR: 34–66 years) for adult patients and two years (IQR: 0.3–11 years) for paediatric patients. The median time from procedure to infection onset was 19 days (IQR: 8–40 days). More than half of CSF shunt SSIs (54.4%, n=56/103) were identified from new surgeries, while 45.6% (n=47/103) were from revision surgeries. Women represented 46.6% (n=48/103) of cases.

**Figure 4:**
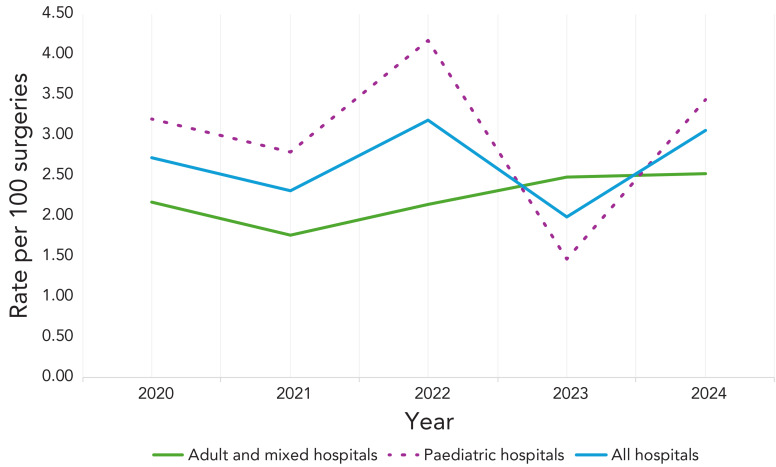
Cerebrospinal fluid shunt surgical site infection rates per 100 surgeries by hospital type^a^, 2020–2024 ^a^ All hospitals include adult, mixed and paediatric hospitals participating in cerebrospinal fluid shunt surgical site infection surveillance

**Trends over time:** The overall rate of CSF shunt SSIs was 2.64 infections per 100 surgeries (range: 1.99–3.19 infections per 100 surgeries; Appendix, **Table A3**). Paediatric and adult/mixed hospitals infection rates were not significantly different at 3.07 and 2.18 infections per 100 surgeries, respectively (*p*=0.15). From 2020 to 2024, no significant trend was observed in CSF shunt SSI rates for adult and mixed hospitals (range: 1.76–2.53 infections per 100 surgeries, *p*=0.88), paediatric hospitals (range: 1.47–4.18 infections per 100 surgeries, *p*=0.11) and all hospital types combined (*p*=0.50) (**Figure 4**). The most common pathogens identified from CSF shunt SSIs were CoNS (29.7%) and *S. aureus* (28.8%) (Table 3). Outcome data were not collected for CSF shunt SSI surveillance.

### Paediatric cardiac surgical site infections

**Infection characteristics:** Between 2020 and 2024, a total of 199 paediatric cardiac SSIs were reported (**Table 6**). Most infections were superficial incisional SSIs (68.8%), followed by organ/space infections (21.1%) and deep incisional infections (10.1%). The median patient age was 69 days (IQR: 8–365 days) and the median time from surgery to infection onset was 14 days (IQR: 8–22 days) (data available on request). The proportion of deep incisional infections increased from 5.4% in 2020 to 15.4% in 2023, followed by a decrease to 8.0% in 2024; however, the increase observed between 2020 and 2023 was not significant (*p*=0.09, Table 6).

**Table 6:** Paediatric cardiac surgical site infection rates by year and infection type, 2020–2024

Year	Superficial incisional SSI cases		Organ/space SSI cases		Deep incisional SSI cases		All cases^a^
	n	%	n	%	n	%	n
2020	29	78.4%	6	16.2%	2	5.4%	37
2021	23	65.7%	9	25.7%	3	8.6%	35
2022	16	64.0%	6	24.0%	3	12.0%	25
2023	32	61.5%	12	23.1%	8	15.4%	52
2024	37	74.0%	9	18.0%	4	8.0%	50
Overall	137	68.8%	42	21.1%	20	10.1%	199

Abbreviation: SSI, surgical site infection

^a^ Excludes cases with missing infection type information

**Figure 5:**
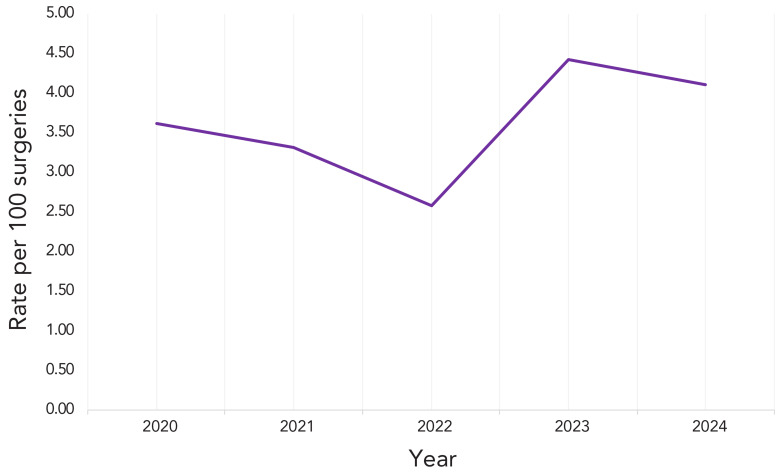
Paediatric cardiac surgical site infection rates per 100 surgeries, 2020–2024

**Trends over time:** The overall paediatric cardiac SSI rate was 3.61 infections per 100 surgeries, with annual rates fluctuating between 2.59 and 4.43 infections per 100 surgeries (**Figure 5**; Appendix, **Table A4**). No significant trend was observed during this five-year period. From 2020 to 2024, at 30 days post-infection, 70.0% of patients had been discharged. Five deaths (2.5% of cases) were reported within 30 days of infection onset, including one death indirectly attributable to the paediatric cardiac SSI (data available on request). The most common pathogens identified among paediatric cardiac SSIs were *S. aureus* (59.0%) and CoNS (14.1%).

### Antibiogram

**Figure 6:**
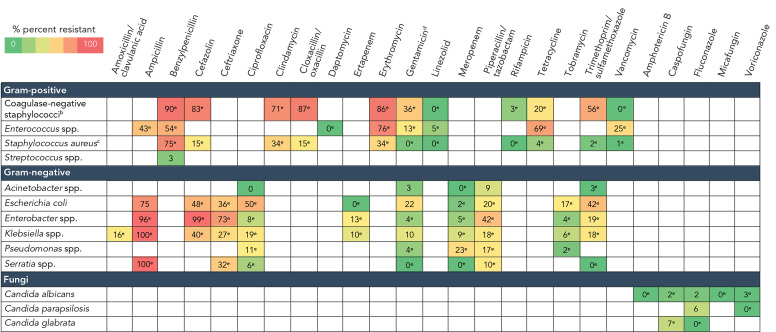
Antibiogram results^a^ from pathogens identified from intensive care unit central line-associated bloodstream infections, 2020–2024^b,c,d,e^ ^a^ Antibiotic/organism combinations with fewer than 30 tests were excluded ^b^ Coagulase-negative staphylococci included *S. lugdunensis*, *S. haemolyticus*, *S. epidermidis*, *S. capitis*, *S. hominis* and *S. warneri* ^c^ *Staphylococcus aureus* included methicillin-susceptible *S. aureus* and methicillin-resistant *S. aureus* (MRSA) ^d^ Gentamicin synergy for gram-positive organisms ^e^ Less than 90% of isolates were tested

**Figure 7:**
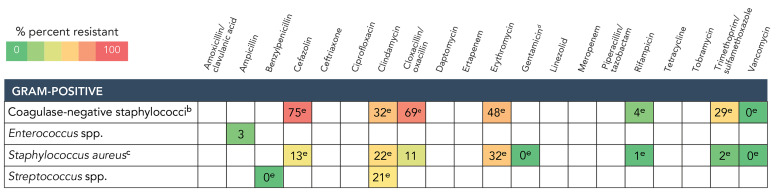
Antibiogram results^a^ from pathogens identified from hip and knee, cerebrospinal fluid shunt and paediatric cardiac surgical site infections, 2020–2024^b,c,d,e^ ^a^ Antibiotic/organism combinations with fewer than 30 tests were excluded ^b^ Coagulase-negative staphylococci included *S. lugdunensis*, *S. haemolyticus*, *S. epidermidis*, *S. capitis*, *S. hominis* and *S. warneri* ^c^ *Staphylococcus aureus* included methicillin-susceptible *S. aureus* and methicillin-resistant *S. aureus* (MRSA) ^d^ Gentamicin synergy for gram-positive organisms ^e^ Less than 90% of isolates were tested

Results of antimicrobial susceptibility testing for the most frequently identified gram-positive, gram-negative and fungal pathogens from device and surgical procedure-related HAIs are listed in **Figure 6** and **Figure 7**. The *S. aureus* isolates were resistant to cloxacillin/oxacillin (MRSA) in 15.1% (n=32/212) of ICU-CLABSIs and 11.1% (n=41/370) of SSIs. Meropenem resistance ranged from 0% to 23% in gram-negative pathogens identified from ICU-CLABSIs. No meropenem resistance was observed among pathogens isolated from SSIs. Ninety-seven vancomycin-resistant *Enterococci* were identified among ICU-CLABSIs (24.8%, n=97/391).

## Discussion

Between 2020 and 2024, there were up to 67 unique hospitals submitted device (ICU-CLABSI) and surgical procedure-related infection data, including 1,846 ICU-CLABSIs and 1,014 surgical procedure-related infections. A total of 3,111 pathogens were identified from ICU-CLABSIs and 1,072 pathogens from surgical procedure-related cases between 2020 and 2024. Similar to what has been reported in past CNISP surveillance, reports (10–12), the majority of these pathogens were gram-positive and the most commonly reported pathogens were coagulase-negative staphylococci and *S. aureus*.

### Intensive care unit central line-associated bloodstream infections

Overall rates of CLABSIs in adult ICUs from 2020 to 2024 (1.89 and 0.97 infections per 1,000 line days in adult mixed ICUs and CVICUs, respectively) were lower than the ICU-CLABSI rates reported from adult ICUs in England during the same period (ranging from 1.8 to 3.6 infections per 1,000 line days) (13). Conversely, CNISP adult ICU CLABSI rates and were higher than quarterly rates reported from 12 adult ICUs in Western Australia (range: 0–0.63 infections per 1,000 line days) (14). Comparisons made between CNISP data and those from other jurisdictions should be interpreted with caution, as data collection is not standardized; therefore, there may be differences in the two populations being compared.

The incidence of CLABSIs in paediatric and neonatal ICUs reported via CNISP was higher compared to England, where rates decreased from fiscal years 2020 to 2024 (1.0 and 0.8 infections per 1,000 line days, respectively) (10). Neonatal ICU-CLABSI rates reported in South Korea from 2020–2022 were 12% lower compared to rates reported by CNISP during the same time period (1.39 vs. 1.58 infections per 1,000 line days) (15).

Differences in catheter utilization ratios likely contributed to the observed variation in CLABSI burden across ICU settings, with higher catheter utilization ratios in adult ICUs (66%–87%) compared to the lowest in the NICU (28%–30%). Thirty-day all-cause mortality was highest in adult ICUs (32%–33%), consistent with greater baseline severity, while paediatric and neonatal mortality remained substantially lower (9.7%–11%), representing different population risk profiles across ICU settings. Thirty-day all-cause mortality was highest in adult ICUs (32%–33%), consistent with greater baseline severity, while paediatric and neonatal mortality remained substantially lower (9.7%–11%). Day-all-cause mortality was highest in adult ICUs (32%–33%), consistent with greater baseline severity, while paediatric and neonatal mortality remained substantially lower (9.7%–11%).

### Surgical site infections

**Hip and knee surgical site infections:** Hip SSI rates fluctuated across reporting years, while knee SSI rates increased non-significantly from 2020 and 2023, before decreasing again in 2024. Hip and knee SSI rates demonstrate ongoing variation across jurisdictions. Long term trends from England’s National Health Service hospitals show a continued steady decline in inpatient hip and knee SSI incidence over the past decade from 2015 to 2025 (16). From 2020 to 2024, hip SSI rates in Western Australia were higher than those reported by CNISP (0.75 vs. 0.64 infections per 100 surgeries), while knee SSI rates were lower (0.29 vs. 0.36 per 100 surgeries) (14).

**Cerebrospinal fluid shunt surgical site infections:** The overall rate of SSIs from CSF shunts was 2.65 per 100 surgeries from 2020 to 2024. The rate is similar to what was previously reported by CNISP from 2019 to 2023 (2.89 per 100 surgeries); however, differences may reflect changes in hospital participation (two fewer hospitals participated in reporting CSF shunt SSIs in 2024 at the time of this study) (10). A national survey conducted in England in 2017 reported a mean brain shunt infection rate of 1.9% (range: 0%–4.4%), which is lower than the overall rate reported by CNISP (17). In contrast, a retrospective single-center study in Sweden reported a higher shunt infection rate of 4.8% in adult hydrocephalus patients who underwent surgery between 2013 and 2019 (18).

Cerebrospinal fluid shunt infection rates vary widely by population (paediatric vs adult), centre and country (18). Combined with a lack of recent literature, comparisons of the data in this report with other regions is limited; therefore, CSF shunt SSIs reported during this time period were compared with previously published CNISP surveillance data (10,11,19). Consistent with earlier findings, CSF shunt SSI rates fluctuated from 2020 to 2024 (10,11,19). The rates observed from 2020 to 2024 were lower than those reported in earlier periods (2000–2002) for both paediatric (3.1% vs 4.9%) and adult patients (2.2% vs 3.2%), similar to the most recent published report (10,19).

**Paediatric cardiac surgical site infections:** From 2020 to 2024, CNISP hospitals reported an overall paediatric cardiac SSI rate of 3.7 infections per 100 surgeries, with no significant trend observed over the five-year period. Due to a lack of published national reports from other jurisdictions, these data could not be compared directly with other countries. Single-center studies from several jurisdictions have observed varied rates of paediatric cardiac SSIs: from 0.9 infections per 100 surgeries in a California, United States centre (20) to 1.97 in a single centre in Ontario, Canada from 2022 to 2023 (21), to 4.34 in a centre in Chile from 2015 to 2020 (22). However, these studies may not be representative of the entire nation. When compared to previously published CNISP surveillance data, rates of paediatric cardiac SSIs have remained relatively unchanged since data collection began in 2010. From 2011 to 2020, the rate of infection was 3.5 per 100 surgeries (11).

### Antibiogram

Meaningful comparisons with other regions are limited by gaps in recent literature and variation in how antibiogram data are reported for device-related and surgical procedure-related infections. To address this, we compared 2020–2024 data with CNISP surveillance data from 2011 to 2020; however, since the time periods overlap, observed changes may not reflect true trends and should be interpreted with caution (11). The percentage of *S. aureus* isolates that were MRSA among ICU-CLABSIs (15%) and SSIs (11%) in the CNISP network remained relatively stable over the 2020–2024 period compared to previous surveillance data from 2011 to 2020, where MRSA accounted for 15% of ICU-CLABSIs and 14% of SSIs. Among *Enterococcus* spp. identified in ICU-CLABSIs, 25% were vancomycin-resistant *Enterococci*, compared to 16% in 2011–2020 (11). During the 2011–2020 time period, results for meropenem resistance in *Pseudomonas* spp. identified in ICU-CLABSIs were not available; however, in later CNISP surveillance reports, a decrease in resistance was observed; from 38% in 2018–2022 to 30% in 2019–2023 to 23% in 2020–2024 (10,12). Meropenem resistance among other gram-negative pathogens identified in ICU-CLABSIs ranged from 0% to 9% in 2020–2024 and from 2% to 7% in 2011–2020 (11).

### Strengths and limitations

The main strength of CNISP surveillance is the standardized collection of detailed epidemiological and molecular-linked data from a large representative network of sentinel hospitals from across Canada. From 2020 to 2024, CNISP coverage of Canadian acute care beds has increased from 35% to 49%, including increased representativeness in northern, community, rural and Indigenous populations. To further improve representativeness, CNISP has launched a simplified dataset accessible to all acute care hospitals across Canada to collect and visualize annual HAI rate data. Despite the increased representativeness of CNISP surveillance data, the number of hospitals participating in each HAI surveillance project differed and epidemiologic data collected were limited to the information available in the patient charts. For CLABSI surveillance, data were limited to infections occurring in the ICU settings and, as such, may represent only a subset of CLABSIs occurring in the hospital. Furthermore, when comparing our infection rates with data from other countries, several limitations must be considered, including differences in surveillance methodologies, patient populations and number and types of hospitals under surveillance.

## Conclusion

This report summarizes 1,846 device-related infections and 1,014 surgical procedure-related infections as well as antibiogram data identified over five years of surveillance, 2020–2024, from up to 67 hospitals across Canada. During this time, rates of device- and surgical procedure-related HAIs have fluctuated from year to year with no significant trend throughout the study period. The collection and analysis of national surveillance data are important to understanding and reducing the burden of device and surgical procedure-related HAIs. These data provide benchmark rates for national and international comparison and inform antimicrobial stewardship and infection prevention and control programs and policies.

## Appendix

Supplemental material is available upon request to the author: cnisp-pcsin@phac-aspc.gc.ca

Case definitions

Table A1: Rate of central line-associated bloodstream infection per 1,000 line days by intensive care unit type, 2020–2024

Table A2: Rate of hip and knee surgical site infections per 100 surgeries, 2020–2024

Table A3: Cerebrospinal fluid shunt surgical site infection rates per 100 surgeries by hospital type, 2020–2024

Table A4: Paediatric cardiac surgical site infection rates per 100 surgeries, 2020–2024
